# High-Order Exponentially Fitted Methods for Accurate Prediction of Milling Stability

**DOI:** 10.3390/mi16090997

**Published:** 2025-08-29

**Authors:** Yi Wu, Bin Deng, Qinghua Zhao, Tuo Ye, Anmin Liu, Wenbo Jiang

**Affiliations:** 1 School of Intelligent Manufacturing and Mechanical Engineering, Hunan Institute of Technology, Hengyang 421002, China; 2005001476@hnit.edu.cn (B.D.); 2017001002@hnit.edu.cn (T.Y.); 2004001114@hnit.edu.cn (A.L.); 2 Hunan Meibeida Technology Co., Ltd., Leiyang 421800, China; 6868@3202778.cn (Q.Z.); jiangwenbo@xdwzc.com (W.J.)

**Keywords:** stability prediction, chatter vibration, multistep schemes, milling operations, implicit exponentially fitted methods

## Abstract

Regenerative chatter is an unfavorable phenomenon that severely affects machining efficiency and surface finish in milling operations. The prediction of chatter stability is an important way to obtain the stable cutting zone. Based on implicit multistep schemes, this paper presents the third-order and fourth-order implicit exponentially fitted methods (3rd IEM and 4th IEM) for milling stability prediction. To begin with, the delay differential equations (DDEs) with time-periodic coefficients are employed to describe the milling dynamics models, and the principal period of the coefficient matrix is firstly decomposed into two different subintervals according to the cutting state. Subsequently, the fourth-step and fifth-step implicit exponential fitting schemes are applied to more accurately estimate the state term. Two benchmark milling models are utilized to illustrate the effectiveness and advantages of the high-order implicit exponentially fitted methods by making comparisons with the three typical existing methods. Under different radial immersion conditions, the numerical results demonstrate that the 3rd IEM and the 4th IEM exhibit both faster convergence rates and higher prediction accuracy than the other three existing prediction methods, without much loss of computational efficiency. Finally, in order to verify the feasibility of the 3rd IEM and the 4th IEM, a series of experimental verifications are conducted using a computer numerical control machining center. It is clearly visible that the stability boundaries predicted by the 3rd IEM and the 4th IEM are mostly consistent with the cutting test results, which indicates that the proposed high-order exponentially fitted methods achieve significantly better prediction performance for actual milling processes.

## 1. Introduction

Machining chatter is commonly perceived as an undesirable self-excited vibration in the modern manufacturing industry and has various negative effects such as poor surface roughness, accelerated tool wear, and low machining productivity. To achieve high performance milling operations, the determination of the stability limit with high accuracy and efficiency is an important strategy for selecting optimal cutting parameters. Regenerative chatter caused by the phase difference is considered to be the most common obstacle for milling processes [1,2,3], and the corresponding mathematical model of milling dynamics can be generally modeled by DDEs [4,5]. To avoid the regenerative chatter, selecting suitable process parameters is of great significance.

The milling dynamic equation can be directly solved by different solution methods to calculate the stability lobe diagram (SLD), and the chatter-free parameter combinations for achieving higher machining productivity can be determined according to the SLD. Up to now, various existing methods, mainly including numerical, analytical, and experimental methods, have been proposed to obtain more accurate and efficient prediction results. These approaches are often referred to as the zeroth-order approximation method (ZOA) [6], multi-frequency solution method (MFS) [7], temporal finite element analysis method (TFEA) [8], semi-discretization method (SDM) [9,10], Chebyshev collocation method (CCM) [11], full-discretization method (FDM) [12], enhanced multistage homotopy perturbation method (EMHPM) [13], complete discretization scheme (CDS) [14], Runge–Kutta-based complete discretization method (RKCDM) [15], precise integration method (PIM) [16], holistic-discretization method (HDM) [17], numerical differentiation method (NDM) [18], numerical integration method (NIM) [19], Simpson-based method (SBM) [20], Adams–Moulton-based method (AMM) [21], and implicit exponentially fitted method (IEM) [22]. Altintas et al. [6] transformed the original DDEs into frequency-domain components and creatively proposed an efficient ZOA method by merely retaining the zero-order term of Fourier series expansion. This method can achieve satisfactory calculation accuracy under large radial immersion milling conditions due to the critical axial cutting depth being expressed analytically in an explicit form, but it is unsuitable for small radial immersion milling. Merdol et al. [7] considered the greater number of harmonics terms and further developed the MFS method to solve the main limitation of the ZOA method. Unlike analytical methods, Bayly et al. [8] suggested the TFEA method by utilizing the cubic Hermite polynomials to calculate the tool vibration displacement. Insperger et al. [9,10] adopted two different order piecewise constant functions to approximate the delay term of DDEs and presented the zeroth-order and first-order SDMs (0th SDM and 1st SDM) based on the direct difference scheme (DDS). Subsequently, the SDMs have been widely expanded to predict the stability of various machining operations [23,24]. By approximating the exponential matrices with precise integration, Jiang et al. [25] and Yan et al. [26] further developed higher-order SDMs for accurately and efficiently generating the stability lobes, in which the higher-order interpolation polynomials were utilized. Based on the properties of Chebyshev polynomials, Butcher and his co-authors [11] recommended the CCM through selecting the optimal collocation points. On this basis, Totis et al. [27] extended the CCM for variable spindle speed milling.

Applying the direct integration scheme (DIS), Ding et al. [12] applied the well-accepted first-order FDM (1st FDM) to solve the Duhamel term of the precise response, which has relatively high calculation speed compared with the 1st SDM without sacrificing numerical accuracy. Based on higher-order interpolation polynomials, the second-order FDM [28], third-order FDM [29], third-order Hermite approximation method [30], Hermite–Newton approximation method [31], least squares approximation methods [32], and high-order vector numerical integration methods [33] were further introduced for improving the prediction accuracy. Nevertheless, the structures of these extension algorithms are becoming progressively more complex with the improvement of the interpolation order, resulting in low computational speed. Olvera et al. [13] and Sosa et al. [34] presented the EMHPM by using a different order polynomial. With the aid of different high-order polynomials, various updated versions of FDMs (UFDMs) were successively introduced by Tang et al. [35] and Yan et al. [36] for reducing the computational time, in which the state transition matrixes were established directly. In the framework of UFDM, Ma et al. [37] reported a novel update FDM by estimating the state term with cubic spline interpolation. Liu et al. [38] suggested an update FDM (E-FDM) by using the fourth-order Lagrange interpolation to calculate the time-delay term. By discretizing all parts of DDEs with Euler’s method, Li et al. [14] reported the CDS to further enhance the computational speed. Xie [39] extended the CDS method and recommended the improved CDS (ICDS) by estimating periodic coefficient matrices with linear interpolation. Based on the Lagrange polynomial and Euler’s formula, Xia et al. [40] put forward the numerical integral Euler’s method (NIEM) for more efficient stability prediction of milling. Li et al. [15] introduced the RKCDM by using the high-order numerical method, in which the stability boundaries are determined by dichotomy search for higher efficiency. Niu and co-workers [41] brought forward the generalized Runge–Kutta method (GRKM) by combining the fourth-order Runge–Kutta and Simpson methods. Dai et al. [16] and Li et al. [42] suggested the two precise integration methods (PIM and IPIM) by using the different-order Taylor formula to expand the nonhomogeneous term. Numerical results demonstrate that the convergence rate of the IPIM is much faster than that of the PIM. Dai and his co-authors [43] developed the generalized precise integration method (GPIM) for the chatter stability of five-axis ball-end milling. With the aid of the holistic-interpolation scheme, Qin et al. [17,44] presented two HDMs (HDM and PCHDM) through approximating the integral nonhomogeneous term with second-order Lagrange interpolation. On the basis of finite difference formulas, Zhang et al. [18] reported the NDM for prediction of stability lobes in high-speed milling. Ding et al. [19] and Dong et al. [45] recommended the so-called NIMs for milling stability prediction based on numerical integration formulas. Li et al. [46] adopted the Newton–Cotes rules to deduce the numerical solutions and developed the Newton–Cotes-based method (NCM) for obtaining higher computational efficiency. Zhang and co-workers [20] studied stability lobe diagrams using the SBM, which has a relatively higher calculation speed compared with the 1st SDM and 2nd FDM. To achieve higher convergence rates, Qin et al. [21,47] suggested the AMM and Chebyshev-wavelet-based methods for stability prediction of milling operations. Zhi et al. [48] introduced the implicit Adams method for milling analysis, in which the state term was approximated by the linear multi-step method. Based on Fibonacci search, Wu et al. [22] presented the 2nd IEM for determining the milling stability lobes with single and multiple delays. In terms of the truncation error, the high-order exponentially fitted methods have the obvious advantage of an extremely small error constant. However, it is worth noting that the state term has not been estimated by the high-order exponentially fitted methods.

To enhance the convergence rate and prediction accuracy, the third-order and fourth-order implicit exponentially fitted methods are utilized to approximate the state term. Numerical simulations and verification experiments are carried out to demonstrate the availability of the 3rd IEM and the 4th IEM. The remainder of this paper is organized as follows. Section 2 presents the third-order and fourth-order implicit exponentially fitted methods. Section 3 examines the effectiveness of the 3rd IEM and the 4th IEM through detailed comparisons with the 2nd SDM, the CCM, and the 2nd IEM. Section 4 performs the various experimental verifications. Section 5 gives the main conclusions of this paper.

## 2. Dynamics Modeling of Milling Operations and Numerical Algorithms

Based on the regenerative chatter theory, the dynamic mathematical model of the milling system can be formulated as the following DDE.

(1) $$M\overset{..}{q}\left(t\right)+C\dot{q}\left(t\right)+\mathrm{Kq}\left(t\right)=-a_{p}K_{c}(t)\left[q\left(t\right)-q\left(t-T\right)\right],$$

where the matrixes M, C, and K denote the modal parameters of cutting tool. Simultaneously, $a_{p}$ and $q\left(t\right)$ represent the axial depth of the cut and the modal displacement vector of the cutter, respectively. Based on the two-DOF milling model, $q\left(t\right)$ indicates the displacement vector in both x and y directions. *T* stands for the principal period of the system that can be computed by T=60/(NΩ), where *N* represents the number of cutter teeth and Ω denotes the rotation speed of the spindle (rpm). The matrix $K_{c}(t)$ stands for the cutting force coefficient satisfying $K_{c}(t)=K_{c}(t+T)$.

Without a loss of generality, the milling dynamics Equation (1) can be directly converted into the following state-space form as

(2) $$\overset{\cdot }{U}(t)=A_{0}U(t)+A(t)\left[U(t)-U(t-T)\right]$$

where

(3) $$A_{0}=\left[\begin{array}{cc} -M^{-1}C/2 & M^{-1}\\ {\mathrm{CM}}^{-1}C/4-K & -M^{-1}C/2 \end{array}\right],A\left(t\right)=\left[\begin{array}{cc} 0 & 0\\ -a_{p}K_{c}(t) & 0 \end{array}\right]$$

Based on the DIS [12], Equation (2) is deduced as follows:

(4) $$U(t)=e^{A_{0}\left(t-t_{0}\right)}U(t_{0})+\int_{t_{0}}^{t}e^{A_{0}(t-\xi )}A(\xi )[U(\xi )-U(\xi -T)]d\xi$$

where $t_{0}$ represents the initial time point.

According to the existence of any non-zero elements of the coefficient matrix, the milling vibration form can be directly determined. Based on the above analysis, the principal period of the system can be split into the free and forced vibration phases in [19,21]. When all the elements of the matrix $K_{c}(t)$ are zero, it suffers from the free vibration operation; Equation (4) can be analytically deduced as

(5) $$U(t)=e^{A_{0}\left(t-t_{0}\right)}U(t_{0})$$

In this work, we divide equally the forced vibration phase into *m* very short time intervals with the step length of *h*. Then, the corresponding discretized time nodes $t_{n}\left(i=1,2,\cdots ,m+1\right)$ can be given by

(6) $$t_{n}=t_{0}+t_{f}+\left(n-1\right)h$$

During the time span $\left[t_{n},t_{n+1}\right]$, Equation (4) at arbitrary time nodes $t_{n}$ can be equivalently re-written as

(7) $$U(t)=e^{A_{0}\left(t-t_{n}\right)}U(t_{n})+\int_{t_{n}}^{t}e^{A_{0}(t-\xi )}A(\xi )[U(\xi )-U(\xi -T)]d\xi$$

### 2.1. Third-Order Implicit Exponentially Fitted Method (3rd IEM)

To construct the state transition matrix, the implicit exponentially fitted methods are applied to solve the Equation (7). To simplify the derivation process, the nodal values $U(t_{n})$, $U(t_{n-T})$, and $A(t_{n})$ are denoted as $U_{n}$, $U_{n-T}$, and $A_{n}$, respectively. Based on the fourth-step implicit exponential fitting scheme, the value $U_{n+1}$ can be obtained as follows:

(8) $$\begin{array}{ll} U_{n+1} & ={\mathrm{DU}}_{n}+h(D_{0}+D_{1}+D_{2}+D_{3})A_{n+1}\left[U_{n+1}-U_{n+1-T}\right]-h(D_{1}+2D_{2}+3D_{3})A_{n}\left[U_{n}-U_{n-T}\right]\\ & +h(D_{2}+3D_{3})A_{n-1}\left[U_{n-1}-U_{n-1-T}\right]-hD_{3}A_{n-2}\left[U_{n-2}-U_{n-2-T}\right] \end{array}$$

where

(9) $$D=e^{A_{0}h}$$

(10) $$D_{0}=-\frac{A_{0}^{-1}}{h}(I-D)$$

(11) $$D_{1}=-\frac{A_{0}^{-1}}{h}D-{\left(\frac{A_{0}^{-1}}{h}\right)}^{2}(I-D)$$

(12) $$D_{2}=-{\left(\frac{A_{0}^{-1}}{h}\right)}^{2}(\frac{1}{2}I+\frac{1}{2}D)-{\left(\frac{A_{0}^{-1}}{h}\right)}^{3}(I-D)$$

(13) $$D_{3}=-{\left(\frac{A_{0}^{-1}}{h}\right)}^{2}(\frac{1}{3}I+\frac{1}{6}D)-{\left(\frac{A_{0}^{-1}}{h}\right)}^{3}I-{\left(\frac{A_{0}^{-1}}{h}\right)}^{4}(I-D)$$

Then, Equation (8) can be further rewritten as follows:

(14) $$P_{n-2}U_{n-2}+P_{n-1}U_{n-1}+P_{n}U_{n}+P_{n+1}U_{n+1}=Q_{n-2}U_{n-2-T}+Q_{n-1}U_{n-1-T}+Q_{n}U_{n-T}+Q_{n+1}U_{n+1-T}$$

where

(15) $$\left\{\begin{array}{l} P_{n-2}=hD_{3}A_{n-2},P_{n-1}=-h(D_{2}+3D_{3})A_{n-1}\\ P_{n}=-D+h(D_{1}+2D_{2}+3D_{3})A_{n},P_{n+1}=I-h(D_{0}+D_{1}+D_{2}+D_{3})A_{n+1}\\ Q_{n-2}=hD_{3}A_{n-2},Q_{n-1}=-h(D_{2}+3D_{3})A_{n-1}\\ Q_{n}=h(D_{1}+2D_{2}+3D_{3})A_{n},Q_{n+1}=-h(D_{0}+D_{1}+D_{2}+D_{3})A_{n+1} \end{array}\right.$$

Moreover, the two-step and three-step implicit exponential fitting schemes are used to approximate $U_{2}$ and $U_{3}$ as follows:

(16) $$M_{1}U_{1}+M_{2}U_{2}=N_{1}U_{1-T}+N_{2}U_{2-T}$$

where

(17) $$\left\{\begin{array}{l} M_{1}=-D+hD_{1}A_{1},M_{2}=I-h(D_{0}+D_{1})A_{2}\\ N_{1}=hD_{1}A_{1},N_{2}=-h(D_{0}+D_{1})A_{2} \end{array}\right.$$

(18) $$K_{1}U_{1}+K_{2}U_{2}+K_{3}U_{3}=L_{1}U_{1-T}+L_{2}U_{2-T}+L_{3}U_{3-T}$$

where

(19) $$\left\{\begin{array}{l} K_{1}=-hD_{2}A_{1},K_{2}=-D+h(D_{1}+2D_{2})A_{2},K_{3}=I-h(D_{0}+D_{1}+D_{2})A_{3}\\ L_{1}=-hD_{2}A_{1},L_{2}=h(D_{1}+2D_{2})A_{2},L_{3}=-h(D_{0}+D_{1}+D_{2})A_{3} \end{array}\right.$$

By combining Equations (5), (14), (16) and (18), a linear discrete map can be defined as

(20) $$E_{1}\left[\begin{array}{c} U_{1}\\ U_{2}\\ \vdots \\ U_{m}\\ U_{m+1} \end{array}\right]=F_{1}\left[\begin{array}{c} U_{1-T}\\ U_{2-T}\\ \vdots \\ U_{m-T}\\ U_{m+1-T} \end{array}\right]$$

where

(21) $$E_{1}=\left[\begin{array}{ccccc} I & & & & \\ M_{1} & M_{2} & & & \\ K_{1} & K_{2} & K_{3} & & \\ P_{1} & P_{2} & P_{3} & P_{4} & \\ \ddots & \ddots & \ddots & \ddots & \\ & P_{m-2} & P_{m-1} & P_{m} & P_{m+1} \end{array}\right]$$

and

(22) $$F_{1}=\left[\begin{array}{ccccc} & & & & e^{A_{0}t_{f}}\\ N_{1} & N_{2} & & & \\ L_{1} & L_{2} & L_{3} & & \\ Q_{1} & Q_{2} & Q_{3} & Q_{4} & \\ \ddots & \ddots & \ddots & \ddots & \\ & Q_{m-2} & Q_{m-1} & Q_{m} & Q_{m+1} \end{array}\right]$$

The state transition matrix $\Psi_{1}$ with the third-order implicit exponentially fitted method (3rd IEM) can be determined as

(23) $$\Psi_{1}={\left(E_{1}\right)}^{-1}F_{1}$$

Based on the Floquet theory, when the all eigenvalues of $\Psi_{1}$ are less than one, the linear periodic system is asymptotically stable.

### 2.2. Fourth-Order Implicit Exponentially Fitted Method (4th IEM)

In a similar way, the implicit exponentially fitted methods are utilized to solve Equation (7). Based on the fifth-step implicit exponentially fitted scheme, the value $U_{n+1}$ can be described as:

(24) $$\begin{array}{ll} U_{n+1} & ={\mathrm{DU}}_{n}+h(D_{0}+D_{1}+D_{2}+D_{3}+D_{4})A_{n+1}\left[U_{n+1}-U_{n+1-T}\right]\\ & -h(D_{1}+2D_{2}+3D_{3}+4D_{4})A_{n}\left[U_{n}-U_{n-T}\right]+h(D_{2}+3D_{3}+6D_{4})A_{n-1}\left[U_{n-1}-U_{n-1-T}\right]\\ & -h(D_{3}+4D_{4})A_{n-2}\left[U_{n-2}-U_{n-2-T}\right]+hD_{4}A_{n-3}\left[U_{n-3}-U_{n-3-T}\right] \end{array}$$

where

(25) $$\begin{array}{ll} D_{4}= & -{\left(\frac{A_{0}^{-1}}{h}\right)}^{2}(\frac{1}{4}I+\frac{1}{12}D)-{\left(\frac{A_{0}^{-1}}{h}\right)}^{3}(\frac{11}{12}I+\frac{1}{12}D)\\ & -{\left(\frac{A_{0}^{-1}}{h}\right)}^{4}(\frac{3}{2}I-\frac{1}{2}D)-{\left(\frac{A_{0}^{-1}}{h}\right)}^{5}(I-D) \end{array}$$

Then, Equation (24) can be further rewritten as follows:

(26) $$\begin{array}{ll} R_{n-3}U_{n-3} & +R_{n-2}U_{n-2}+R_{n-1}U_{n-1}+R_{n}U_{n}+R_{n+1}U_{n+1}\\ & =S_{n-3}U_{n-3-T}+S_{n-2}U_{n-2-T}+S_{n-1}U_{n-1-T}+S_{n}U_{n-T}+S_{n+1}U_{n+1-T} \end{array}$$

where

(27) $$\left\{\begin{array}{l} R_{n-3}=-hD_{4}A_{n-3},R_{n-2}=h(D_{3}+4D_{4})A_{n-2},R_{n-1}=-h(D_{2}+3D_{3}+6D_{4})A_{n-1}\\ R_{n}=-D+h(D_{1}+2D_{2}+3D_{3}+4D_{4})A_{n},R_{n+1}=I-h(D_{0}+D_{1}+D_{2}+D_{3}+D_{4})A_{n+1}\\ S_{n-3}=-hD_{4}A_{n-3},S_{n-2}=h(D_{3}+4D_{4})A_{n-2},S_{n-1}=-h(D_{2}+3D_{3}+6D_{4})A_{n-1}\\ S_{n}=h(D_{1}+2D_{2}+3D_{3}+4D_{4})A_{n},S_{n+1}=-h(D_{0}+D_{1}+D_{2}+D_{3}+D_{4})A_{n+1} \end{array}\right.$$

By combining Equations (5), (14), (16), (18) and (26), a linear discrete map can be defined as

(28) $$E_{2}\left[\begin{array}{c} U_{1}\\ U_{2}\\ \vdots \\ U_{m}\\ U_{m+1} \end{array}\right]=F_{2}\left[\begin{array}{c} U_{1-T}\\ U_{2-T}\\ \vdots \\ U_{m-T}\\ U_{m+1-T} \end{array}\right]$$

where

(29) $$E_{2}=\left[\begin{array}{cccccc} I & & & & & \\ M_{1} & M_{2} & & & & \\ K_{1} & K_{2} & K_{3} & & & \\ P_{1} & P_{2} & P_{3} & P_{4} & & \\ R_{1} & R_{2} & R_{3} & R_{4} & R_{5} & \\ \ddots & \ddots & \ddots & \ddots & \ddots & \\ & R_{m-3} & R_{m-2} & R_{m-1} & R_{m} & R_{m+1} \end{array}\right]$$

and

(30) $$F_{2}=\left[\begin{array}{cccccc} & & & & & e^{A_{0}t_{f}}\\ N_{1} & N_{2} & & & & \\ L_{1} & L_{2} & L_{3} & & & \\ Q_{1} & Q_{2} & Q_{3} & Q_{4} & & \\ S_{1} & S_{2} & S_{3} & S_{4} & S_{5} & \\ \ddots & \ddots & \ddots & \ddots & \ddots & \\ & S_{m-3} & S_{m-2} & S_{m-1} & S_{m} & S_{m+1} \end{array}\right]$$

The state transition matrix $\Psi_{2}$ with the fourth-order implicit exponentially fitted method (4th IEM) can be determined as

(31) $$\Psi_{2}={\left(E_{2}\right)}^{-1}F_{2}$$

**Figure 1:**
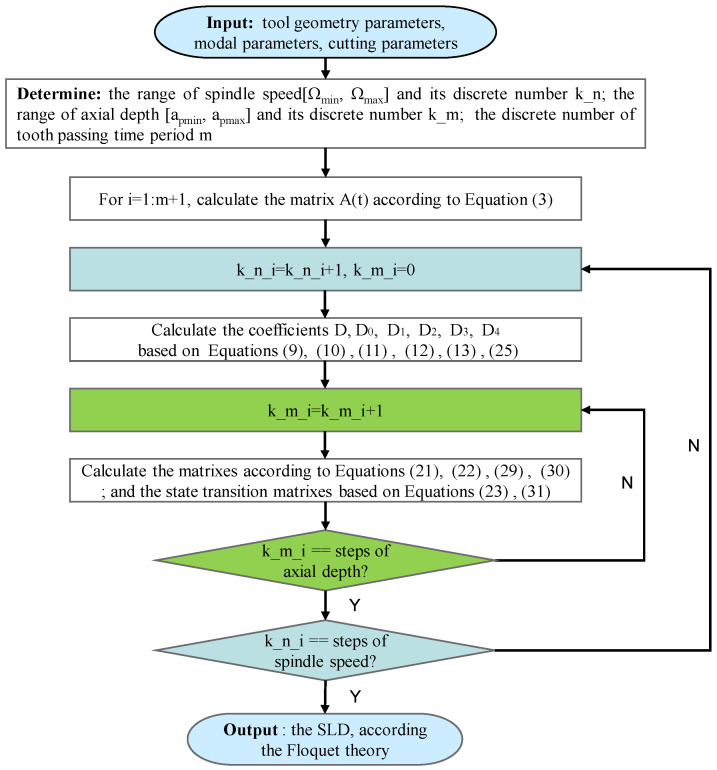
Flow chart for determining the SLD of milling operations using the 3rd IEM and the 4th IEM.

Based on the Floquet theory, when the all eigenvalues of $\Psi_{2}$ are less than one, the linear periodic system is asymptotically stable. On the basis of the two proposed IEMs for stability analysis, the flow chart for determining the SLD is exhibited as Figure 1.

## 3. Numerical Analysis and Discussion

In this section, the two benchmark milling models and machining parameters from Refs. [10,19] are commonly adopted to evaluate the effectiveness of the high-order exponentially fitted methods based on implicit multistep schemes, and the values of the machining parameters are listed in Table 1. Comparisons are made among the 2nd SDM, the CCM, and the IEMs in terms of convergence rate and computational efficiency for algorithm validation. Meanwhile, the stability boundaries of these two models are also calculated by utilizing the 2nd SDM, the CCM, and the IEMs under the same computational conditions.

### 3.1. Convergence Rate Verification

The local discretization error (LDE) is commonly defined as the difference between the reference spectral radius $\left|\mu_{0}\right|$ and the approximate spectral radius $\left|\mu \right|$, where $\left|\mu_{0}\right|$ and $\left|\mu \right|$ are the exact and predicted critical eigenvalues of the state transition matrix, respectively. Mathematically, the LDE can be taken as an important evaluation criterion to estimate the computational accuracy of numerical methods. The convergence rate describes how fast the LDE gradually converges to zero as the discrete number *m* increases. Generally, if the stability prediction method exhibits a higher convergence rate, the LDE will approach zero more quickly. As described in the literature [25], the LDE of the 2nd SDM could run up to $O(h^{3})$, where *h* indicates the discretization step. Following a similar approach, it has been demonstrated in Ref. [22] that the LDE for the 2nd IEM can be ascertained as $O(h^{5})$. Ref. [11] shows that the CCM has spectral convergence accuracy. Based on Ref. [49], the 3rd IEM and the 4th IEM have fifth-order and sixth-order algebraic precision, respectively. Similar to the 2nd IEM, it can be found that the LDEs of IEM with the 3rd and 4th in this paper can be determined as $O(h^{6})$ and $O(h^{7})$, respectively. Precisely for this reason, the proposed high-order exponentially fitted methods have the potential to achieve significantly faster convergence rates than the other three existing prediction methods. In order to further prove the above results, the convergence rates of the proposed IEMs are validated through the single-DOF milling model and comparisons with the 2nd SDM, the CCM, and the 2nd IEM.

**Figure 2:**
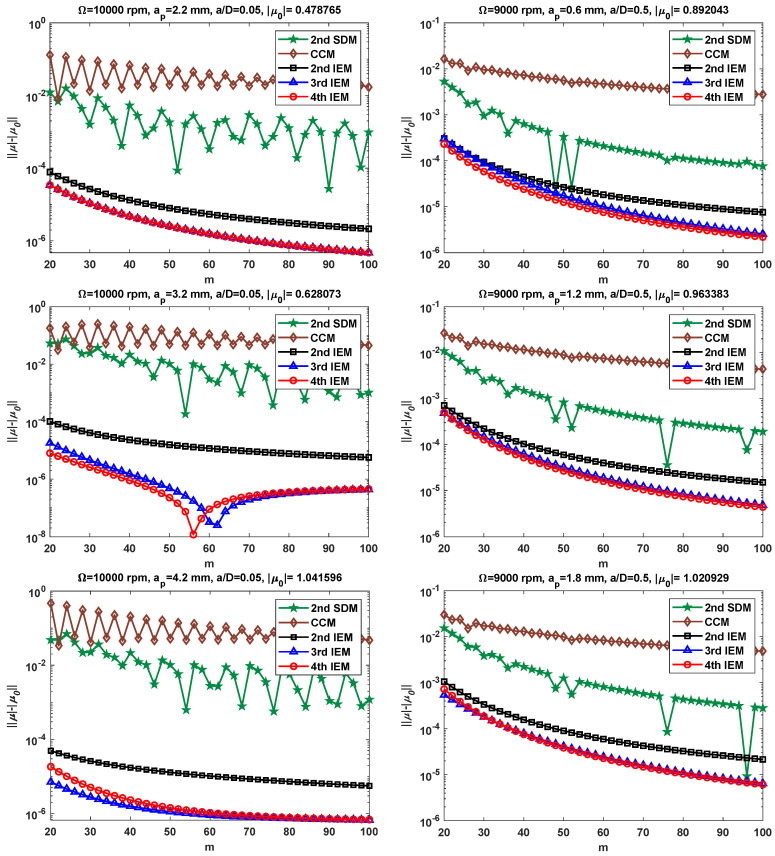
Comparison of convergence rates for the 2nd SDM, the CCM, the 2nd IEM, the 3rd IEM, and the 4th IEM with small and middle radial immersion ratios.

Based on single-DOF milling system, various machining parameter combinations are selected to evaluate the convergence effect of the proposed IEMs more objectively. To begin with, the down milling operations with two radial immersion ratios a/D = 0.05 and 0.5 are examined. Simultaneously, two different spindle speeds and six critical axial depths of cut are used for the verification of the proposed IEMs, i.e., Ω = 10,000 rpm, $a_{p}$ = 2.2, 3.2, and 4.2 mm; and Ω = 9000 rpm, $a_{p}$ = 0.6, 1.2, and 1.8 mm. It is worth noting that the $\left|\mu_{0}\right|$ in this paper is determined by using the 2nd IEM with the large discrete number *m* = 1000, while the $\left|\mu \right|$ is calculated by the 2nd SDM, the CCM, the 2nd IEM, the 3rd IEM, and the 4th IEM under different discrete numbers *m*. Because the LDE of the 2nd IEM is much smaller than the other two existing methods under the same discrete number, $\left|\mu_{0}\right|$ is regarded as the exact value. Figure 2 illustrates the convergence rates for the 2nd SDM, the CCM, the 2nd IEM, the 3rd IEM, and the 4th IEM with six groups of milling parameters.

As shown in Figure 2, the 3rd IEM and the 4th IEM converge to the exact value more quickly than the 2nd SDM, the CCM, and the 2nd IEM with the increase in the *m* value. For example, when *m* = 50 and $a_{p}$ = 3.2 mm, the LDEs acquired by the 2nd SDM, the CCM, the 2nd IEM, the 3rd IEM, and the 4th IEM are 1.02 × $10^{-2}$, 4.92 × $10^{-2}$, 1.56 × $10^{-5}$, 4.78 × $10^{-7}$, and 2.26 × $10^{-7}$, respectively. Additionally, it is easily noticed that the 2nd SDM and the CCM suffer violent oscillations. The IEMs exhibit smooth convergence behavior based on the convolution integral term, which indicates that the proposed IEMs are obviously superior to the 2nd SDM and the CCM in terms of numerical stability. Compared with the 3rd IEM, it can be seen from Figure 2 that the 4th IEM converges a little faster in five different cases, which usually means that the 4th IEM requires less computation time to achieve higher prediction accuracy. However, when the spindle speed Ω = 10,000 rpm and the axial depth of cut $a_{p}$ = 4.2 mm, it is clear that the convergence rate of the 3rd IEM is only slightly higher than that of the 4th IEM. Hence, the proposed IEMs acquire faster convergence and better precision than the other existing methods for both small and middle radial immersion conditions.

**Figure 3:**
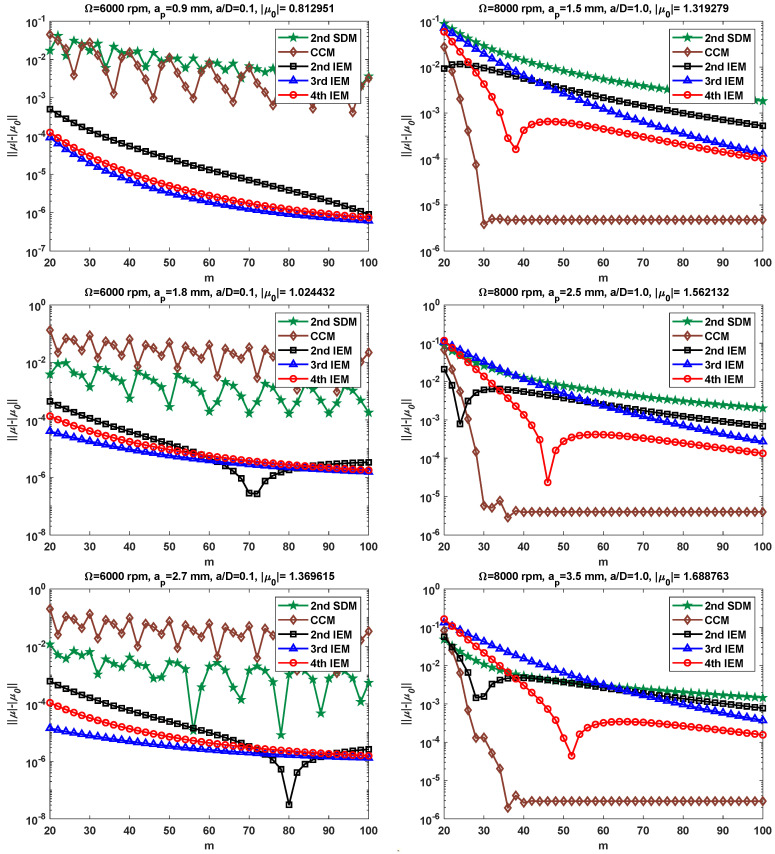
Comparison of convergence rates for the 2nd SDM, the CCM, the 2nd IEM, the 3rd IEM, and the 4th IEM with low and large radial immersion ratios.

To further assess the convergence effect of the proposed IEMs, various combinations of machining parameters include two spindle speeds Ω = 6000 and 8000 rpm; six types of axial depths of cut $a_{p}$ = 0.9, 1.8, 2.7, 1.5, 2.5, and 3.5 mm; and two radial immersion ratios a/D = 0.1 and 1.0, which are selected for verification. Figure 3 illustrates the convergence rates of the 2nd SDM, the CCM, the 2nd IEM, the 3rd IEM, and the 4th IEM with low and large radial immersion conditions. The reference spectral radius $\left|\mu_{0}\right|$ is still determined by using the 2nd IEM with *m* = 1000. It can be seen from Figure 3 that the convergence rates of the 3rd IEM and the 4th IEM are significantly better than those of the 2nd SDM, the CCM, and the 2nd IEM when the spindle speed Ω is set as 6000 rpm and the radial immersion ratio a/D is chosen as 0.1, while the convergence rate of the 3rd IEM is slightly faster than that of the 4th IEM. Meanwhile, when the spindle speed Ω = 8000 rpm and the radial immersion ratio a/D = 1.0, the 4th IEM converges to the exact value much faster than the 2nd SDM, the 2nd IEM, and the 3rd IEM, while the CCM achieves higher accuracy than the 2nd SDM, the 2nd IEM, the 3rd IEM, and the 4th IEM. Moreover, it is clearly found that the IEMs, including the 2nd IEM, the 3rd IEM, and the 4th IEM, exhibit superior numerical stability compared to the 2nd SDM under two different radial immersion conditions. All in all, Figure 2 and Figure 3 validate that the 3rd IEM and the 4th IEM achieve higher convergence precision and numerical stability than the other three methods for different radial immersion ratios and spindle speeds.

### 3.2. Stability Lobes Prediction and Comparison

#### 3.2.1. Single-DOF Milling Operation

**Figure 4:**
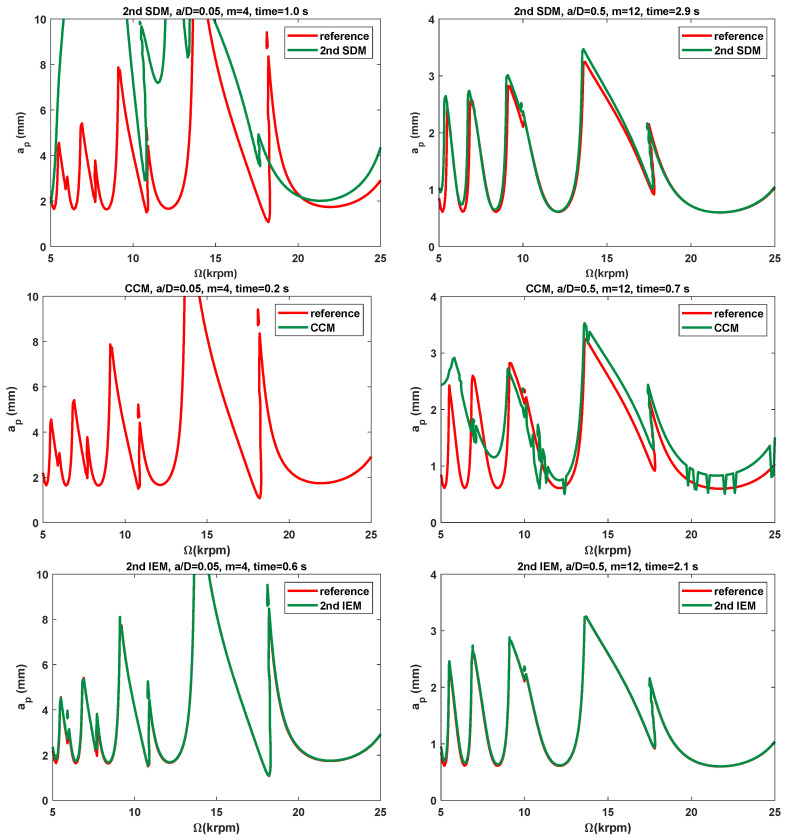

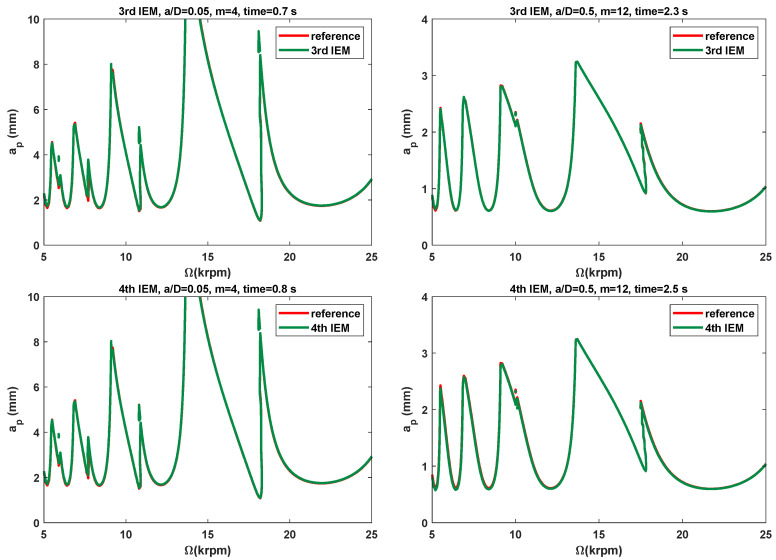
The SLDs predicted by the 2nd SDM, the CCM, the 2nd IEM, the 3rd IEM, and the 4th IEM with a/D = 0.05 and 0.5.

**Figure 5:**
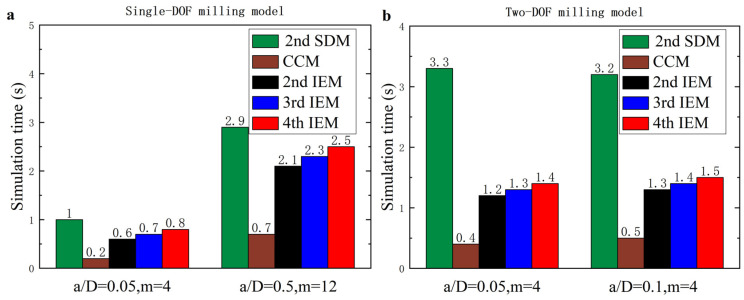
Comparisons of computational time consumed by the 2nd SDM, the CCM, the 2nd IEM, the 3rd IEM, and the 4th IEM. (**a**) Single-DOF milling model, (**b**) Two-DOF milling model.

To evaluate the effectiveness of the proposed IEMs and examine the low and medium radial immersion milling, the 2nd SDM, the CCM, the 2nd IEM, the 3rd IEM, and the 4th IEM are applied directly to construct the SLDs over a 200×100-sized equidistance grid. Based on the single-DOF milling model, the SLDs and corresponding computational times of these five methods with the discrete number *m* are, respectively, chosen as 4 and 12 and are shown in Figure 4 and Table 2. Here, two different radial immersion ratios a/D = 0.05 and 0.5 and the domain of machining parameters are selected to generate the SLDs. As exhibited in Figure 4, the reference stability boundaries represented by the red solid line are determined by the 4th IEM with a relatively large discrete number *m* = 800. Meanwhile, the prediction stability boundaries represented by the green solid line are calculated via the three existing methods and the proposed IEMs under the same discrete number. It is indicated from Figure 4 that the stability boundaries predicted by the proposed IEMs are more consistent with the reference stability boundaries, the stability boundaries predicted by the 2nd IEM exhibit only small deviations from the reference stability boundaries, and the stability boundaries predicted by the other two existing methods reveal noticeable deviations from the reference stability boundaries, which means that the prediction accuracy of the SLDs with the proposed IEMs are much better than those computed by the 2nd SDM, the CCM, and the 2nd IEM under low and medium radial immersion conditions. Moreover, it can be clearly seen from Table 2 that when the radial immersion ratio a/D = 0.5 and discrete number *m* = 12, the 2nd SDM, the CCM, the 2nd IEM, the 3rd IEM, and the 4th IEM consume 2.9 s, 0.7 s, 2.1 s, 2.3 s, and 2.5 s to generate the SLDs, respectively. In comparison with the 2nd SDM, Figure 5 shows the calculation time of the 3rd IEM and the 4th IEM can be reduced by nearly 21% and 14%, respectively. This demonstrates that the proposed IEMs achieve faster computing speed than the 2nd SDM under the same conditions. Simultaneously, the calculation times of the 3rd IEM and the 4th IEM are almost similar to that of the 2nd IEM. Therefore, the 3rd IEM and the 4th IEM obtain better prediction accuracy with a small loss of computational efficiency.

**Figure 6:**
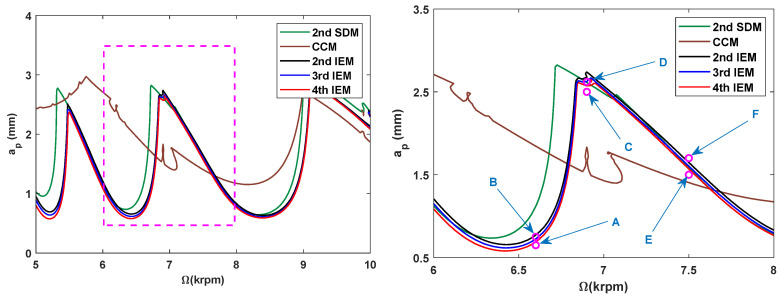
Stability boundaries obtained by different methods over spindle speed changes from 5 to 10 krpm for a/D=0.5.

Based on the marginal stability conditions, the predictive accuracy of the 4th IEM is further validated by using the MATLAB 2021a routine dde23. Numerical simulations are performed by the proposed IEMs and the other three existing methods with a/D = 0.5 and *m* = 12, and the predicted SLDs are obtained over spindle speed changes from 5 to 10 krpm to distinguish the milling stability boundaries. Figure 6 presents the obtained SLDs and their local subgraph over a 200×100 sized equidistance grid. To investigate the computational accuracy of the 2nd SDM, the CCM, the 2nd IEM, the 3rd IEM, and the 4th IEM, the time responses of six marginal parameter points A (6600 rpm, 0.65 mm), B (6600 rpm, 0.75 mm), C (6900 rpm, 2.50 mm), D (6900 rpm, 2.62 mm), E (7500 rpm, 1.50 mm), and F (7500 rpm, 1.70 mm) are calculated numerically to judge whether these points are in a stable or unstable state.

**Figure 7:**
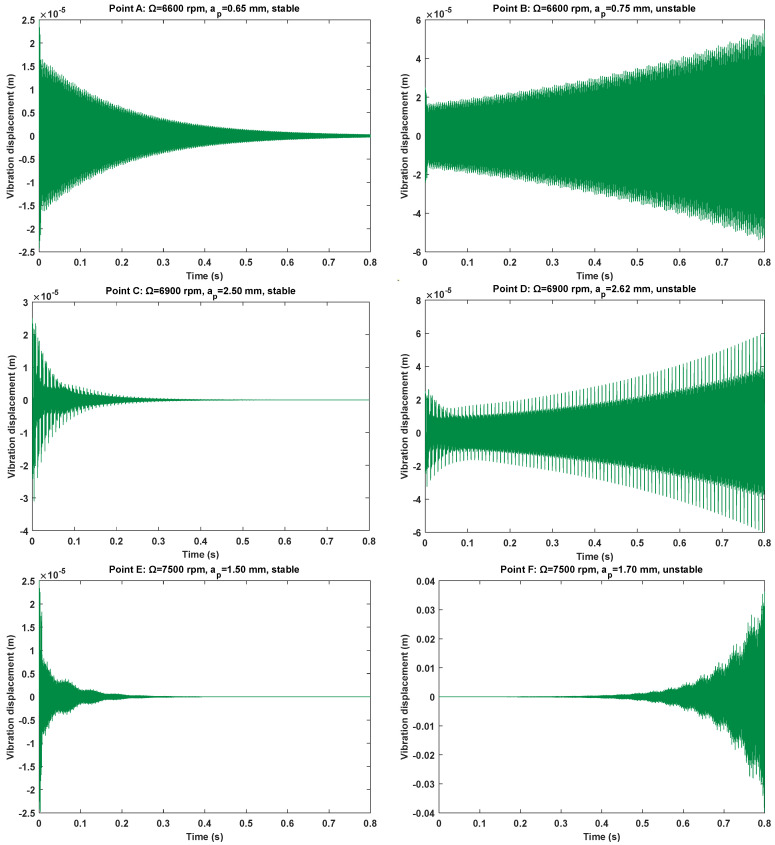
Vibration displacement responses of six marginal parameter points A, B, C, D, E, and F.

The maximal solver step size and relative tolerance are chosen as $10^{-5}$, and initial condition is set as (25 μm, 0 m/s) to investigate the vibration displacement responses of six marginal parameter points, as shown in Figure 7. It is seen in the low-speed region that three groups of parameter points A, C, and E are stable, while the other three groups of parameter points B, D, and F are unstable. This indicates that stability curves are within the axial depths of cut ranges from 0.65 mm to 0.75 mm when the spindle speed is 6600 rpm; the axial depths of cut ranges from 2.50 mm and 2.62 mm when the spindle speed is 6900 rpm, and the axial depths of cut ranges from 1.50 mm and 1.70 mm when the spindle speed 7500 rpm. All the predicted cutting states in the 2nd SDM and the CCM are completely incorrect. The cutting states of points B and D are certainly misjudged in the 2nd IEM, and the cutting states of point D are certainly misjudged in the 3rd IEM. Nevertheless, the cutting states are basically consistent with the predicted results of the 4th IEM. Consequently, the numerical results demonstrate that the accuracy of stability boundaries with the 4th IEM is much better than those computed by the 2nd SDM, the CCM, the 2nd IEM, and the 3rd IEM for marginal stability conditions.

#### 3.2.2. Two-DOF Milling Operation

**Figure 8:**
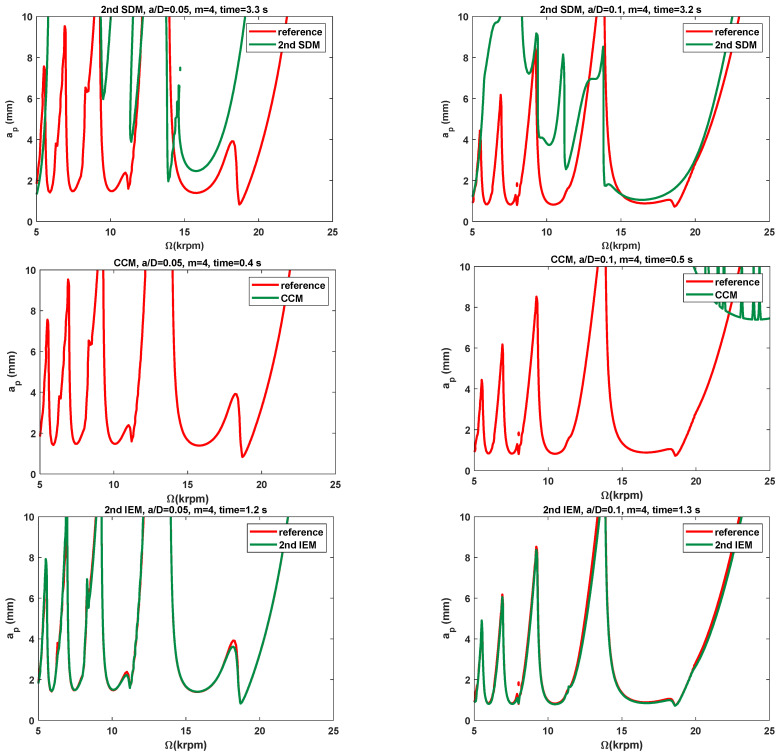

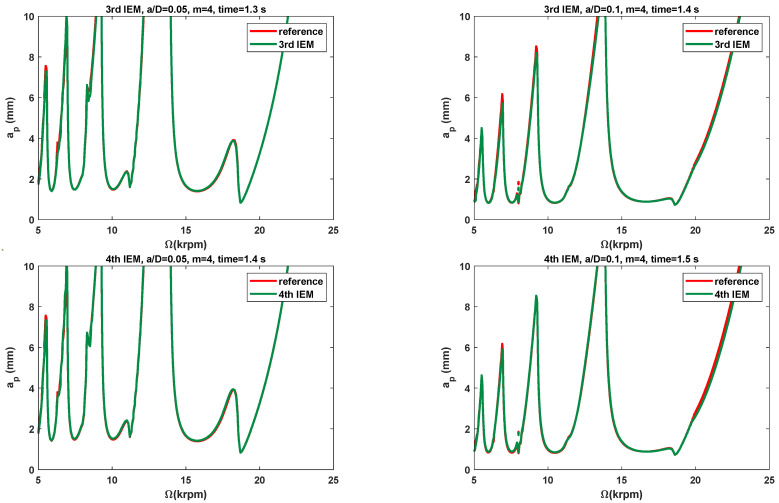
The SLDs predicted by the 2nd SDM, the CCM, the 2nd IEM, the 3rd IEM, and the 4th IEM with a/D = 0.05 and 0.1.

The modal parameters of the two-DOF milling dynamic system and the three conventional methods are employed for further illustrating the effectiveness of the proposed IEMs. To examine the low radial immersion conditions, the 2nd SDM, the CCM, the 2nd IEM, the 3rd IEM, and the 4th IEM are applied directly to construct the predicted SLDs over a 200×100 sized equidistance grid. Based on the two-DOF milling model, the stability lobe diagrams and corresponding computational times of these five methods with the discrete number *m* are chosen as 4 and are exhibited in Figure 8 and Table 2. Now, two different low radial immersion ratios a/D = 0.05 and 0.1 are also utilized to generate the SLDs. As depicted in Figure 8, the reference stability boundaries represented by the red solid line are also computed by the 4th IEM with a sufficiently large discrete number *m* = 800. At the same time, the prediction stability boundaries represented by the green solid line are calculated by the three existing methods and the proposed IEMs under the same discrete number *m*.

It is seen from Figure 8 that the stability boundaries predicted by the proposed IEMs are mostly consistent with the reference stability boundaries, the stability boundaries predicted by the 2nd IEM exhibit only small deviations from the reference stability boundaries, and the stability boundaries predicted by the other two existing methods reveal significant deviations from the reference stability boundaries, which indicates that the prediction accuracy of the SLDs with the proposed IEMs is obviously higher than that computed by the 2nd SDM, the CCM, and the 2nd IEM under small radial immersion conditions. In addition, it can be clearly observed from Table 2 that when the radial immersion ratio a/D = 0.1 and discrete number *m* = 4, the 2nd SDM, the CCM, the 2nd IEM, the 3rd IEM, and the 4th IEM take 3.2 s, 0.5 s, 1.3 s, 1.4 s, and 1.5 s to construct the SLDs, respectively. When compared with the 2nd SDM, Figure 5 reveals the calculation time of the 3rd IEM and the 4th IEM can be saved by approximately 56% and 53%, respectively. It indicates that the proposed IEMs achieve higher efficiency than the 2nd SDM under the same computational conditions. The calculation speeds of the 3rd IEM and the 4th IEM are nearly comparable to that of the 2nd IEM. Therefore, the 3rd IEM and the 4th IEM are proved to have better performances for predicting milling stability lobes.

## 4. Experimental Verification and Analysis

### 4.1. Experimental Verification Based on Benchmark Example

**Figure 9:**
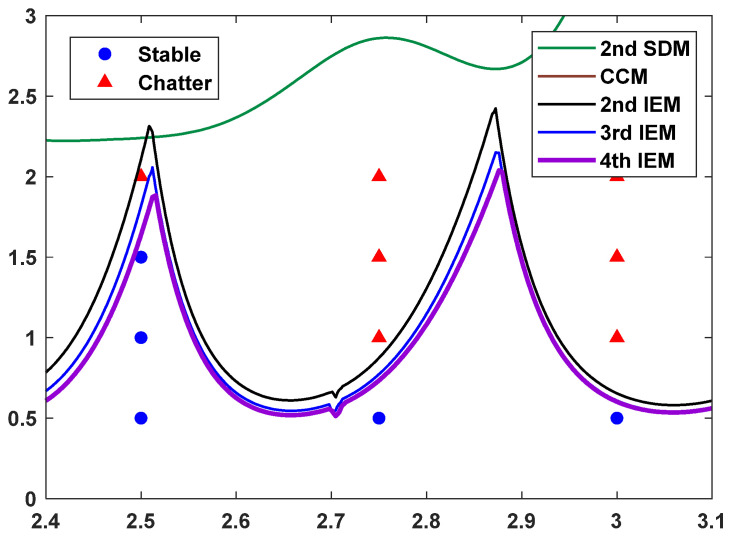
The predicted SLDs using the 2nd SDM, the CCM, the 2nd IEM, the 3rd IEM, and the 4th IEM and actual cutting tests results in low-speed milling.

With the aim of validating the effectiveness of the proposed IEMs, a series of down-milling experiments were conducted on a vertical boring and milling machining center (VF-3). According to Ref. [42], a two-tooth cutter tool with diameter *D* = 10 mm was installed on the machine tool spindle and was also used for cutting tests on the workpiece material of 45 steel. During the milling experiments, the model parameters of the tool tip are identified by impact tests, and the cutting force coefficients are calibrated by slot milling based on linear regression analysis. The modal parameters of the machining system and the cutting force coefficients are displayed in Table 3. The SLDs with the radial immersion ratio a/D = 0.2 and discrete number *m* = 7 are constructed by using the 2nd SDM, the CCM, the 2nd IEM, the 3rd IEM, and the 4th IEM, and twelve groups of cutting tests from the existing literature [42] are exhibited in Figure 9.

As depicted in Figure 9, the twelve observation points can be divided into five stable points and seven unstable points. The red solid triangle represents the fact that the experimental result is unstable, and the blue solid circle represents the fact that the experimental result is stable.

It can be seen from Figure 9 that the stability boundaries predicted by the 2nd SDM and the CCM are completely inconsistent with the cutting test results; the stability boundary predicted by the 2nd IEM reveals slightly deviated from the cutting test results; and the stability boundaries predicted by the 3rd IEM and the 4th IEM exhibit excellent agreement with the cutting test results under small immersion conditions, which indicates that the 3rd IEM and the 4th IEM, based on implicit multistep schemes, can predict the stability lobes under the very small discrete number *m*. Therefore, the availability and applicability of the 3rd IEM and the 4th IEM are thoroughly verified in low-speed milling operations.

**Figure 10:**
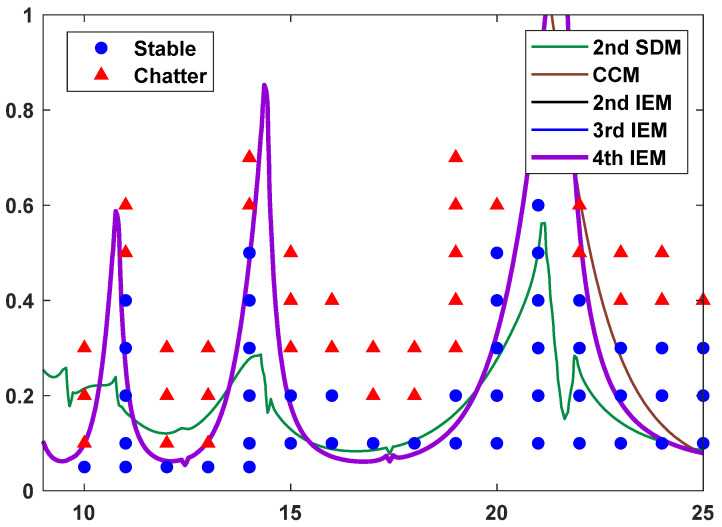
The predicted SLDs using the 2nd SDM, the CCM, the 2nd IEM, the 3rd IEM, and the 4th IEM and actual cutting tests results in high-speed milling.

To further demonstrate the reliability of the proposed IEMs, a series of up-milling experiments is carried out on a high-speed milling center. Based on Ref. [50], a single-tooth cylindrical cutter with diameter *D* = 8 mm was installed on the machine spindle and was also utilized for milling processes on the workpiece material of AlMgSi0.5 aluminum alloy. During high-speed milling operations, the modal parameters of the machining system and the cutting force coefficients are given in Table 4. The SLDs with the radial immersion ratio a/D = 0.5 and discrete number *m* = 7 are constructed by utilizing the 2nd SDM, the CCM, the 2nd IEM, the 3rd IEM, and the 4th IEM, and a large number of parameter combinations are selected, which are exhibited in Figure 10.

To conduct a detailed analysis, it can be seen from Figure 10 that the stability boundaries predicted by the 2nd SDM and the CCM are significantly inconsistent with the cutting test results; the stability boundaries predicted by 2nd IEM, the 3rd IEM, and the 4th IEM are approximately coincident with the cutting test results under medium immersion condition except for a few specific points (caused by experiment measurement error and nonlinear factors), which indicates that the 3rd IEM and the 4th IEM, based on implicit multistep schemes, can predict the stability lobes in the case of high-speed milling. In summary, the 3rd IEM and the 4th IEM have excellent accuracy for predicting the SLDs whether in the case of low-speed or high-speed milling operations.

### 4.2. Experimental Verification Based on Actual Cutting Process

**Figure 11:**
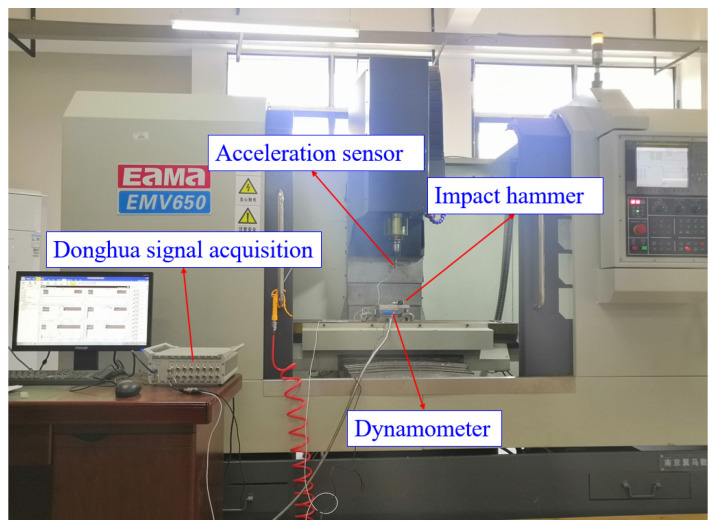
Experimental site for milling operations.

To further evaluate the reliability and feasibility of the proposed high-order exponentially fitted methods, a series of slot milling experiments are performed on a three-axis vertical machining center (EMV650, Yima CNC Machine Tool Co., Ltd., Yima, China). A four-flute end-milling cutter with 100 mm overall length and 10 mm diameter is adopted in all the milling experiments. Meanwhile, the material of the workpiece is the aluminum alloy AL6061. The apparatus for modal parameter determination and cutting force coefficient estimation comprises a Donghua impact hammer LC02 (Jiangsu Donghua Testing Technology Co., Ltd., Taizhou, China), Donghua signal acquisition instrument DH5922 (Jiangsu Donghua Testing Technology Co., Ltd., China), Dytran acceleration sensor 3224A1 (Dytran Instruments, Inc. Chatsworth, CA, USA), and Kistler dynamometer 9257B (Kistler Instrument Corporation, New York, NY, USA), as exhibited in Figure 11. During the impact tests, a Dytran acceleration sensor 3224A1 is utilized to acquire the vibration signals, while a Donghua impact hammer LC02 is used to create the exciting force on the cutter tip of the tool in the x and y directions. The Donghua signal acquisition instrument DH5922 is applied for obtaining the frequency response function and modal analysis. Subsequently, five groups of full slot milling experiments under different feeds per tooth are carried out to identify the cutting force coefficients.

The identified important input parameters are acquired based on the rational fraction polynomials and linear regression results, which are listed in Table 5.

**Figure 12:**
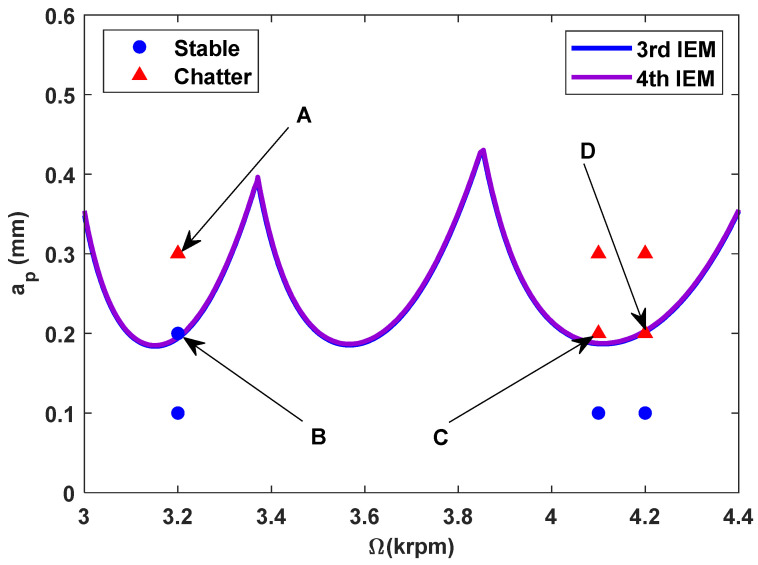
The predicted SLDs established by the 3rd IEM and the 4th IEM.

According to the identified milling parameters, the SLDs calculated by the proposed high-order exponentially fitted methods with the radial immersion ratio a/D = 1.0 and discrete numbers *m* = 100 are displayed in Figure 12.

In the confirmatory experiments, the instantaneous cutting forces are measured by the Kistler dynamometer 9257B with the sampling frequency 10 kHz, and the milling force signals are recorded by the data acquisition HRU-1213MA (Shanghai Haonai Electronic Technology Co., Ltd., Shanghai, China). As shown in Figure 12, under large radial immersion conditions, the stability boundaries predicted by the 3rd IEM and the 4th IEM reveal excellent agreement with the actual experimental results. At the same time, the observed parameter points can be broadly divided into two main categories: one is stable points and the other is unstable points. The red solid triangle is utilized to indicate the cutting state is unstable, and the blue solid circle is applied to indicate the cutting state is stable. To conduct a more thorough study of the milling chatter, four observed parameter combinations, i.e., A (3200 rpm, 0.30 mm), B (3200 rpm, 0.20 mm), C (4100 rpm, 0.20 mm), and D (4200 rpm, 0.20 mm), are selected for executing actual cutting force tests, as exhibited in Table 6. It can be observed from Table 6 that the point B (3200 rpm, 0.20 mm) is stable, and the frequency spectrum of the parameter combination B does not show the chatter frequency; it mainly includes the tooth-passing frequency (3200/60 × 4 = 213 Hz) and its harmonic frequencies. For points A, C, and D, the tooth-passing frequencies are, respectively, 213 Hz, 273 Hz, and 280 Hz, but the chatter frequencies of points A, C, and D occur at (1628 Hz, 1841 Hz), (1595 Hz, 1868 Hz), and (1630 Hz, 1910 Hz), and the difference of these adjacent chatter frequencies is equal to 213 Hz, 273 Hz, and 280 Hz, respectively. Compared with points A, C, and D, the cutting force single of point B is more regular. Obviously, the points A, C, and D can be regarded as unstable cases. According to the above analysis, the proposed high-order exponentially fitted methods can predict the SLDs effectively for the actual milling operations.

## 5. Conclusions

Based on implicit multistep schemes, the third-order and fourth-order implicit exponentially fitted methods (3rd IEM and 4th IEM) are developed in this paper for accurately predicting the milling stability. Some main conclusions are summarized as follows:

(1) To construct the Floquet transition matrix, the principal period of the coefficient matrix is decomposed into two different subintervals, and the fourth-step and five-step implicit exponential fitting schemes are applied to more accurately estimate the state term.
(2) Compared with the three conventional methods, the convergence rates of the high-order exponentially fitted methods are analyzed for the single-DOF milling system. The numerical results demonstrate that the 3rd IEM and 4th IEM achieve much higher convergence rates than the 2nd SDM, the CCM, and the 2nd IEM under different radial immersion conditions.
(3) In comparison with the three existing methods, the SLDs determined by the proposed IEMs are mostly consistent with the reference stability lobes under the identical discrete number. When compared with the 2nd SDM, the calculation time of the 3rd IEM and the 4th IEM can be saved by approximately 56% and 53%, respectively. The calculation speeds of the 3rd IEM and the 4th IEM are nearly comparable to that of the 2nd IEM. Therefore, the 3rd IEM and the 4th IEM are proved to have better performances without sacrificing computational efficiency for predicting milling stability lobes.
(4) The experimental verifications with the two-DOF milling operation demonstrate the applicability and effectiveness of the 3rd IEM and the 4th IEM. The prediction results of the proposed IEMs exhibit excellent agreement with the experimental results, which indicates that the proposed IEMs have the ability to ascertain chatter-free conditions for actual milling processes.

## Figures and Tables

**Table 1:** Milling parameters of the benchmark milling model.

Modal Parameters	Cutting Parameters
$m_{t}$ = 0.03993 kg	$K_{t}$ = $6\times 10^{8}\;N/m^{2}$
ζ = 0.011	$K_{n}$ = $2\times 10^{8}\;N/m^{2}$
$\omega_{n}$ = 922×2π rad/s	*N* = 2

**Table 2:** Calculation time obtained for the 2nd SDM, the CCM, the 2nd IEM, the 3rd IEM, and the 4th IEM (unit: s).

	Models	Single-DOF Milling Model		Two-DOF Milling Model	
Methods		a/D = 0.05, m = 4	a/D = 0.5, m = 12	a/D = 0.05, m = 4	a/D = 0.1, m = 4
2nd SDM		1.0	2.9	3.3	3.2
CCM		0.2	0.7	0.4	0.5
2nd IEM		0.6	2.1	1.2	1.3
3rd IEM		0.7	2.3	1.3	1.4
4th IEM		0.8	2.5	1.4	1.5

**Table 3:** Identified milling parameters.

Modal Parameters	Cutting Parameters
$k_{x}$ = $2.6\times 10^{6}\;N/m$, $k_{y}$ = $1.9\times 10^{6}\;N/m$	$K_{t}$ = $9.197\times 10^{8}\;N/m^{2}$
$\zeta_{x}$ = 0.018, $\zeta_{y}$ = 0.014	$K_{n}$ = $1.399\times 10^{8}\;N/m^{2}$
$\omega_{n}$ = 668×2π rad/s, $\omega_{n}$ = 668×2π rad/s,	*N* = 2

**Table 4:** Milling parameters of the machine-tool system.

Modal Parameters	Cutting Parameters
$k_{x}$ = $4.14\times 10^{5}\;N/m$, $k_{y}$ = $4.09\times 10^{5}\;N/m$	$K_{t}$ = $6.44\times 10^{8}\;N/m^{2}$
$c_{x}$ = 1.56 kg/s, $c_{y}$ = 1.60 kg/s	$K_{n}$ = $0.37\times 6.44\times 10^{8}\;N/m^{2}$
$m_{x}$ = 0.0201 kg, $m_{y}$ = 0.0199 kg	*N* = 1

**Table 5:** Milling parameters of the dynamic system.

Modal Parameters	Cutting Parameters
$\omega_{nx}$ = 1798.096××2π rad/s, $\omega_{ny}$ = 1789.551××2π rad/s	$K_{t}$ = $3.8179\times 10^{8}\;N/m^{2}$
$\zeta_{x}$ = 0.02496, $\zeta_{y}$ = 0.03322	$K_{n}$ = $1.9174\times 10^{8}\;N/m^{2}$
$m_{x}$ = 0.03253 kg, $m_{y}$ = 0.02959 kg	*N* = 4

**Table 6:** Measured cutting force and corresponding frequency spectra of observed parameter points A, B, C, and D.

Group	Time Domain	Frequency Domain
Point A (Chatter)	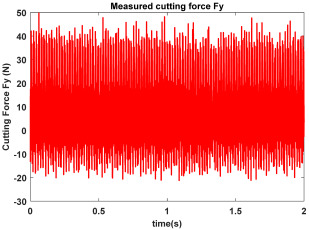	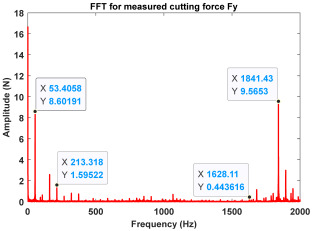
Point B (Stable)	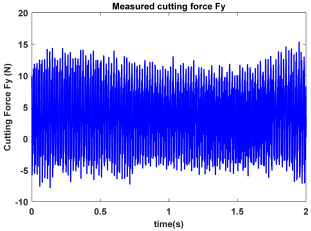	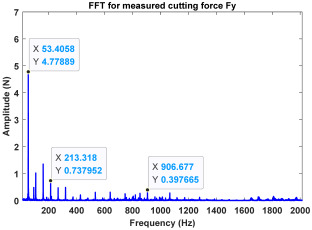
Point C (Chatter))	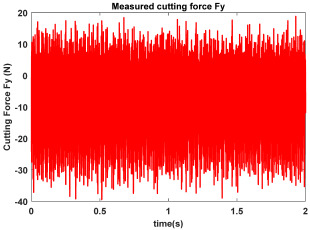	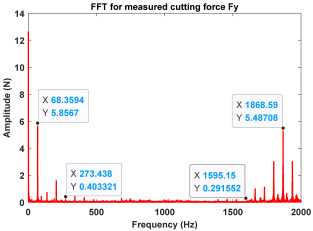
Point D (Chatter)	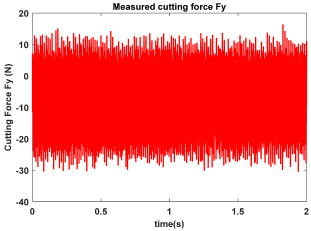	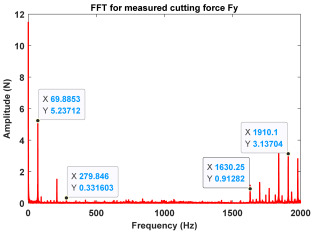

## Data Availability

The original contributions presented in the study are included in the article; further inquiries can be directed to the corresponding author.

## Nomenclature

M	The mass matrix
C	The damping matrix
K	The stiffness matrix
$q\left(t\right)$	The modal displacement vector at the current moment
$q\left(t-T\right)$	The modal displacement vector at the previous tooth-passing period
$a_{p}$	The axial of the depth cut
$K_{c}(t)$	The coefficient matrix
*T*	The principal period of the system
*N*	The number of cutter teeth
Ω	The spindle speed
$t_{f}$	The forced vibration time period
$t_{0}$	The initial time point
$A_{0}$	The constant matrix
Ψ	The state transition matrix
$\left|\mu_{0}\right|$	The reference spectral radius
$\left|\mu \right|$	The approximate spectral radius
*m*	The discrete number
a/D	The radial immersion ratio
$m_{t}$	The modal mass
ζ	The relative damping ratio
c	The damping
*k*	The stiffness
$\omega_{n}$	The angular natural frequency
$K_{t}$	The tangential cutting force coefficient
$K_{n}$	The normal cutting force coefficient
DDE	Delay differential equation
SLD	Stability lobe diagram
DDS	Direct difference scheme
DIS	Direct integration scheme
DOF	Degree of freedom
LED	Local discretization error
